# Targeted antiangiogenic agents in combination with cytotoxic chemotherapy in preclinical and clinical studies in sarcoma

**DOI:** 10.1186/s13569-016-0049-z

**Published:** 2016-06-07

**Authors:** Kieuhoa T. Vo, Katherine K. Matthay, Steven G. DuBois

**Affiliations:** Department of Pediatrics, UCSF School of Medicine, San Francisco School of Medicine, UCSF Benioff Children’s Hospital, University of California, 550 16th Street, 4th Floor, Box 0434, San Francisco, CA 94158 USA; Dana-Farber/Boston Children’s Cancer and Blood Disorders Center, 450 Brookline Avenue, Dana 3, Boston, MA 02215 USA

**Keywords:** Sarcoma, Antiangiogenesis, Combination drug therapy, Combination chemotherapy

## Abstract

Sarcomas are a heterogeneous group of mesenchymal malignancies. In recent years, studies have demonstrated that inhibition of angiogenic pathways or disruption of established vasculature can attenuate the growth of sarcomas. However, when used as monotherapy in the clinical setting, these targeted antiangiogenic agents have only provided modest survival benefits in some sarcoma subtypes, and have not been efficacious in others. Preclinical and early clinical data suggest that the addition of conventional chemotherapy to antiangiogenic agents may lead to more effective therapies for patients with these tumors. In the current review, the authors summarize the available evidence and possible mechanisms supporting this approach.

## Background

Sarcomas are a heterogeneous group of malignancies, including soft tissue sarcomas (STS) and tumors of bone and cartilage. Conventional chemotherapy regimens for advanced or metastatic sarcomas have low survival rates, substantial toxicity, and frequent emergence of resistance, making alternative novel treatment approaches a priority.

Sarcomas express proangiogenic factors that may represent therapeutic targets, with vascular endothelial growth factor (VEGF) being the best characterized. In animal models of human sarcomas, inhibitors of angiogenesis have shown promising antitumor activity [1–3]. Antiangiogenic therapies have a number of potential advantages compared to chemotherapy including overcoming chemoresistance [4, 5], more favorable toxicity profile, and broad spectrum of activity. Since 2004, over ten drugs that target VEGF or its receptors have been approved as cancer therapeutics, with many more in clinical trials [6]. These agents have shown single-agent activity in sarcoma. Most notably, pazopanib has been approved by the US Food and Drug Administration and the European Medicines Agency for advanced STS. As monotherapy, these agents have only provided survival benefits on the order of weeks to months in some sarcoma subtypes, and have not been efficacious in others [7]. Therefore, combining antiangiogenic agents (AA) with other systemic agents active in sarcoma may lead to more effective therapies for patients with these tumors.

This review summarizes evidence supporting the use of targeted AA in combination with cytotoxic chemotherapy in sarcomas. We performed an extensive review of the available medical literature using the US National Library of Medicine’s PubMed search function to find relevant primary articles based on key search terms including “angiogenesis”, “antiangiogenic”, “antiangiogenesis”, and “antivascular”. These search terms were searched with “chemotherapy” and “sarcoma”, “bone tumor”, or “soft tissue cancer”. The “Related Articles” function of PubMed and reference lists from relevant articles were used to identify additional articles. Additionally, in order to identify recent trials not yet published, we also performed a search of abstracts presented at the American Society of Clinical Oncology (ASCO) annual meetings from 2013 to 2015.

In the current review, we provide the results of this search beginning with the preclinical data supporting AA in combination with chemotherapy in this diverse group of diseases. The review concludes with an assessment of the completed and ongoing clinical studies that have treated patients with sarcoma using this therapeutic strategy.

### Preclinical efficacy of targeted AA in combination with chemotherapy

Angiogenesis is tightly regulated at the molecular level. Dysregulation of angiogenesis occurs in various pathologies and is one of the hallmarks of cancer. Concentrated efforts in this area of research are leading to the discovery of a growing number of pro- and anti-angiogenic molecules, many of which are already in clinical trials. The complex interactions among these molecules and how they affect vascular structure and function in different environments are now beginning to be elucidated [6, 8–10]. This integrated understanding is leading to the development of a number of therapeutic approaches to treat cancer, including the use of AA in combination with chemotherapy.

#### Biological mechanisms supporting combination approaches in solid tumor malignancies other than sarcoma

With the discovery of VEGF as a major driver of tumor angiogenesis, efforts have focused on novel therapeutics aimed at inhibiting VEGF activity. Unfortunately, clinical trials of anti-VEGF monotherapy in patients with solid tumors have resulted in only modest responses. Intriguingly, the combination of anti-VEGF therapy with conventional chemotherapy has improved survival in cancer patients compared with chemotherapy alone [6].

**Fig. 1:**
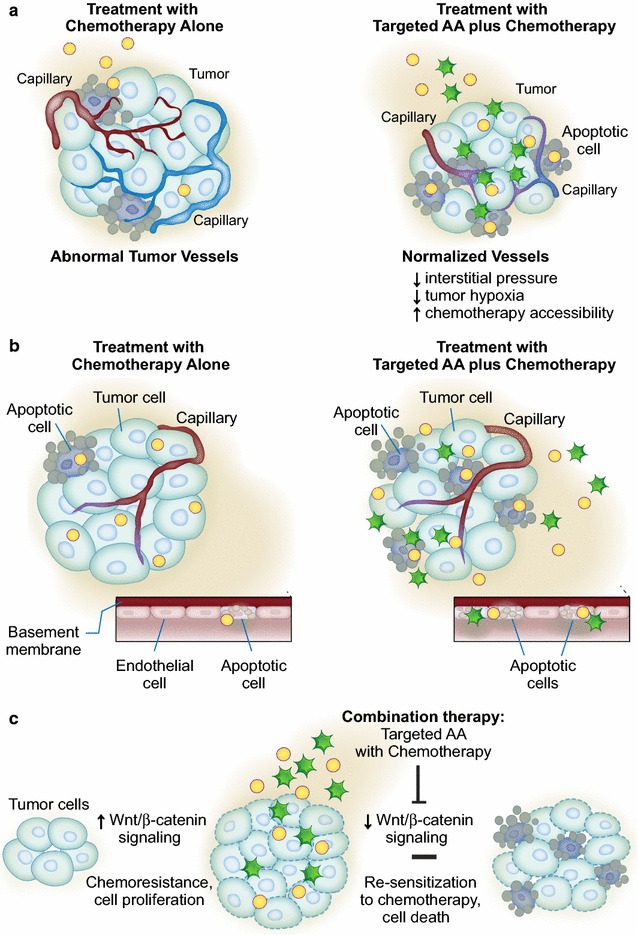
Proposed biological mechanisms supporting combination antiangiogenesis approaches in sarcoma. **a** Transient “normalization” of the abnormal tumor vasculature by AA results in improved blood perfusion and enhanced chemotherapy accessibility and antitumor activity. **b** The synergistic interaction of combination therapy leads to enhanced direct cytotoxicity of tumor cells and/or endothelial cells. **c** Combination therapy leads to up- or down-regulation signaling pathways involved in chemoresistance. For example, down-regulation of the Wnt/β-catenin pathway by the combination of masitinib and gemcitabine contribute to the re-sensitization of gemcitabine-resistant pancreatic tumor cells leading to apoptotic death [27]. *AA* antiangiogenic agents

The proposed mechanisms of benefit from combined AA and chemotherapy include: (1) normalization of the tumor vasculature by altering vascular permeability and increasing drug accessibility (Fig. 1a); (2) synergistic effects leading to enhanced direct cytotoxicity of cancer cells and/or endothelial cells (Fig. 1b); and/or (3) decreased chemoresistance (Fig. 1c).

A paradoxical hypothesis that may explain the antitumor effect of this combination approach relies on the theory of transient “normalization” of the abnormal tumor vasculature, which results in improved blood perfusion and enhanced chemotherapy accessibility and antitumor activity (Fig. 1a) [6]. Several preclinical studies using direct and indirect AA support the normalization hypothesis [11–13]. Blockade of VEGF signaling results in transient pruning and active remodeling of the immature and leaky blood vessels of tumors in animal models so that it more closely resembled the normal vasculature. Functional improvements accompany these morphological changes, including decreased interstitial fluid pressure (IFP), decreased tumor hypoxia, and improved penetration of macromolecules from these vessels into tumors [11–13].

Based on this hypothesis, Liu and colleagues examined the vascular density and structural changes of tumors obtained from lung cancer xenograft mice treated with bevacizumab combined with gemcitabine and cisplatin [14]. They demonstrated significant reduction in VEGF levels and microvessel density (MVD) and increased number of normal vessels as analyzed by electron microscopy in mice treated with combination therapy compared to those mice treated with chemotherapy alone [14]. The tumor volume of mice in the combined treatment group was significantly lower compared to the bevacizumab monotherapy and chemotherapy groups, which also correlated with significant survival advantage [14].

Improved chemotherapy delivery secondary to tumor vessel normalization was demonstrated in a study of bevacizumab and topotecan in neuroblastoma xenograft models. After a single bevacizumab dose, there were decreases in tumor MVD, tumor vessel permeability, and tumor IFP compared to controls [15]. Intratumoral perfusion, as assessed by contrast-enhanced ultrasonography, was also improved [15]. Moreover, intratumoral drug delivery accompanied these changes: penetration of topotecan was improved when given 1–3 days after bevacizumab, compared to concomitant administration or 7 days apart, and resulted in greater tumor growth inhibition than with monotherapy or concomitant administration of the two drugs [15]. Similarly, the increase in antitumor activity of chemotherapy during the transient vascular normalization period produced by bevacizumab has also been confirmed in animal models of colorectal cancer (irinotecan) [16] and melanoma (melphalan) [17].

*In vivo* [(15)O]H2O positron emission tomography (PET) imaging in a mouse model of lung cancer showed that treatment with the VEGFR/platelet-derived growth factor receptor (PDGFR) inhibitor PTK787 created a 7-day window of improved tumor blood flow when tumor vessels are transiently normalized [18]. An improvement in pericyte coverage and reduced leakiness from tumor vessels in xenografts accompanied this normalization phase [18]. Initiation of newer targeted agents during this window of vessel normalization also resulted in increased drug delivery and apoptotic efficacy of erlotinib, an epidermal growth factor receptor (EGFR) inhibitor [18]. Together, these findings offer strong supportive evidence that strategic administration of AA can promote transient vessel normalization that improves drug delivery and efficacy in a range of solid tumors.

In contrast, a study by Van der Veldt et al. in non-small cell lung cancer (NSCLC) showed that pretreatment with bevacizumab reduced both perfusion and net influx rate of radiolabeled docetaxel as measured by PET with effects persisting after 4 days [19]. This study highlighted the importance of drug scheduling and advocated further studies to optimize scheduling of antiangiogenic drugs combined with cytotoxic chemotherapy.

Other preclinical studies reporting the impact of AA upon delivery of cytotoxic therapies include sunitinib, an inhibitor of VEGFR and PDGFR, combined with temozolomide in orthotopic glioma models [20, 21]. Sunitinib significantly increased temozolomide tumor distribution [21]. A “vascular normalization index” incorporating MVD and protein expression of α-SMA and collagen IV was proposed as an indication of the number of tumor vessels with relatively good quality, and significantly correlated with the unbound temozolomide AUC in tumor interstitial fluid [21].

Interestingly, when used as monotherapy, several preclinical studies have shown that the normalization of blood vessels by AA may result in paradoxical increased invasion of local vessels by the tumor and resulting metastases. A recent study of the effects of combination therapy in breast cancer model suggest that the addition of chemotherapy to AA can help prevent local invasion of vessels promoted by the AA and result in lower metastatic rate. Antiangiogenic therapy with DC101 (VEGFR2 inhibitor), while blunting tumor volume growth, was found to increase local invasion in multiple primary tumor models, including a patient-derived xenograft [22]. This effect was blocked by concurrent chemotherapy with paclitaxel [22]. Similarly, the combination of paclitaxel with DC101 caused a marked reduction of micro- or macrometastatic disease in contrast to DC101 monotherapy, which was associated with small increases in metastatic disease.

Synergistic effects of combination therapy of AA with chemotherapy have been seen in several preclinical models of solid cancers (Fig. 1b). For example, in vitro studies of bladder cancer demonstrated the efficacy of pazopanib with docetaxel, even in docetaxel-resistant bladder cancer cell lines [23]. While the mechanism(s) of these synergistic effects have not been fully elucidated, and may be dependent on the specific combination regimen used and tissue type treated, we have highlighted several examples of mechanisms related to enhanced direct cytotoxicity of cancer cells and/or endothelial cells.

Sorafenib increased apoptosis in melanoma-derived cell lines treated with melphalan or temozolomide [24]. The molecular mechanisms underlying sorafenib enhancement were investigated by analyzing the changes in signaling events in melanoma cell lines in response to sorafenib treatment alone. Response to sorafenib correlated with extracellular signal-regulated kinase (ERK) down-regulation and loss of Mcl-1 expression [24]. These results suggest that sorafenib enhanced sensitivity to chemotherapy by altering signaling in the mitogen-activated protein kinase (MAPK) and the mitochondrial apoptotic pathways. These in vitro findings highlight the potential for AA to have effects independent of classical antiangiogenic mechanisms.

The timing and sequence of AA with chemotherapy can also be critical in determination of synergy or antagonism. Troiani et al. demonstrated the sequence-dependent interactions of ZD6474 (VEGR, EGFR, and RET inhibitor) with oxaliplatin in colon cancer cell lines in vitro using three combination schedules [25]. Treatment with oxaliplatin followed by ZD6474 was highly synergistic, whereas the reverse sequence or concurrent exposure was clearly antagonistic [25]. Oxaliplatin induced a G2-M arrest, which was antagonized if the cells were previously or concurrently treated with ZD6474. ZD6474 enhanced oxaliplatin-induced apoptosis, but only when added after oxaliplatin [25].

Alternatively, Naumova and colleagues demonstrated that paclitaxel and SU6668, a VEGFR2/PDGFR inhibitor, synergistically inhibited the proliferation and increased apoptosis of endothelial cells [26]. These findings, together with the in vivo inhibition of angiogenesis in Matrigel plugs and the reduction of MVD of paclitaxel-resistant ovarian carcinoma xenograft models, support the hypothesis that the enhanced effect exerted by the combination of paclitaxel and SU6668 on tumor growth is mediated by an effect on the vasculature [26].

Another mechanism of combination therapy involves overcoming chemoresistance (Fig. 1c). Acquired drug resistance is a major problem in the treatment of cancer. Boehm et al. reported that chronic, intermittent therapy of three different mouse tumors with endostatin, an angiogenic inhibitor, did not show any evidence of acquired drug resistance [5]. In contrast, standard chemotherapy, using maximum doses of cyclophosphamide, resulted in drug resistance in lung carcinoma xenografts [5]. These results provided initial evidence that a specific angiogenic inhibitor does not induce drug resistance in three different tumor xenografts. Perhaps the most significant finding of this study was that repeated cycles of endostatin therapy induced tumor dormancy that persisted after therapy. While the mechanism(s) is not yet clear, recent studies may help to elucidate these findings.

For example, a series of in vitro and in vivo studies using preclinical models of human pancreatic cancer characterized the synergistic effects of combination therapy with gemcitabine with masitinib, a selective inhibitor of PDGFR [27]. The masitinib and gemcitabine combination synergistically inhibited proliferation of gemcitabine-refractory cell lines [27]. Analysis of gene expression profiling of gemcitabine-resistant pancreatic cells revealed differences in gene expression unique to the masitinib plus gemcitabine combination. The most significantly altered pathway involved genes associated with Wnt/β-catenin signaling [27]. This pathway is involved in pancreatic development and re-activation has been implicated in pancreatic carcinoma, suggesting a mechanism of augmented cell death with combination therapy in gemcitabine-resistant cells as compared to gemcitabine monotherapy [27].

#### Preclinical studies of combination approaches in sarcoma

Targeted AA and cytotoxic chemotherapy have been combined in several laboratory models of sarcoma, mainly STS, as summarized in Table 1. Most notably, studies have shown that VEGFR2 blockade by DC101 combined with chemotherapy inhibits tumor growth, metastases, and angiogenesis in STS xenografts [28, 29]. Combined DC101 and continuous low-dose doxorubicin resulted in more effective growth inhibition of STS xenografts compared to either agent alone [28]. DC101 plus doxorubicin also enhanced the inhibition of tumor angiogenesis and endothelial cell activity, as demonstrated by significantly reduced MVD and inhibition of neovascularization [28]. Additionally, this combination regimen directly exerted enhanced inhibitory effects on endothelial cell migration, proliferation, and tube-like formation in vitro. Furthermore, the combination enhanced apoptosis of endothelial cells [28].

**Table 1:** Preclinical studies of combination approaches in sarcoma

Drug combination	Sarcoma tumor models	Results compared to models treated with chemotherapy alone	Reference
Pazopanib + topotecan	OS KHOS and RMS RH30 cell lines and xenografts	↑ Antitumor and antiangiogenic effects, ↑ Survival, ↓ Circulating endothelial cells and/or endothelial progenitor cells, ↓ MVD	[30]
VDA (OXi4503/CA1P) + doxorubicin	EWS xenografts	↑ Antitumor effects ↑ Necrosis ↓ Perfused vasculature	[59]
Bevacizumab + topotecan	ASPS xenografts	↑ Antitumor effects compared to bevacizumab monotherapy, but not topotecan alone	[60]
Vandetanib + doxorubicin	Multiple STS cell lines and xenografts	↑ Antitumor and antiangiogenic effects ↓ Local growth leiomyosarcoma ↓ Lung metastases in fibrosarcoma	[31]
DC101 + doxorubicin	Multiple STS cell lines and xenografts transfected with VEGF_165_	↑ Antitumor and antiangiogenic effects ↓ Tumor growth and pulmonary metastases ↓ MVD ↑ Percentage of mature vessels ↓ Matrix metalloproteinases secreted by endothelial cells	[29]
DC101 + doxorubicin	Leiomyosarcoma SKLMS-1 and RMS RD cell lines and xenografts	↑ Antitumor and antiangiogenic effects ↓ MVD and neovascularization ↑ Apoptosis of endothelial cells ↓ Endothelial cell migration, proliferation, tube-like formation	[28]
TNP-470 + etoposide	Angiosarcoma ISOS-1 cell line and xenograft	↑ Antitumor effects ↑ Growth inhibition	[61]

*ASPS* alveolar soft part sarcoma, *ES* Ewing sarcoma, *MVD* microvessel density, *OS* osteosarcoma, *RMS* rhabdomyosarcoma, *STS* soft tissue sarcoma, *VDA* vascular-disrupting agent, *VEGF(R)* vascular endothelial growth factor (receptor)

To elucidate the role of recombinant human VEGF_165_ in STS growth, metastasis, and chemoresistance, Zhang and colleagues generated stably VEGF_165_-transfected STS cell lines to study the effect of VEGF overexpression in vitro and in vivo. VEGF_165_-transfected xenografts formed highly vascular tumors with shorter latency, accelerated growth, enhanced chemoresistance, and increased incidence of pulmonary metastases [29]. Combined therapy with DC101 and low-dose doxorubicin in vivo suppressed the growth of VEGF_165_-overexpressing xenografts, inhibited angiogenesis, increased the vessel maturation index, and suppressed tumor cell proliferation compared to monotherapy-treated mice. The addition of DC101 induced endothelial cell sensitivity to doxorubicin and suppressed the activity of matrix metalloproteinases secreted by endothelial cells [29]. These results suggested that the antitumor effects of combined therapy with DC101 and doxorubicin were secondary to tumor-associated endothelial cell growth modulation and chemosensitization [29].

Likewise, the enhanced antitumor effects of combination therapy using low-dose topotecan and pazopanib in mouse models of osteosarcoma and rhabdomyosarcoma are thought to be related to augmented antiangiogenesis [30]. The metronomic administration of pazopanib and topotecan in vitro showed reduction in circulating endothelial cells, circulating endothelial progenitor cells, and tumor MVD which correlated with antitumor activity and enhancement in survival compared with monotherapy agents in all preclinical models [30].

Concomitant use of a dual VEGFR2/EGFR inhibitor (vandetanib) with doxorubicin resulted in additional cytotoxicity and endothelial cell growth inhibition with lowered doxorubicin doses compared to vandetanib monotherapy in leiomyosarcoma, fibrosarcoma, and uterine sarcoma models [31]. In addition, vandetanib in combination with low-dose doxorubicin resulted in significant inhibition of human fibrosarcoma xenograft lung metastases compared to control and doxorubicin-only groups [31]. Collectively, these studies suggest that AA plus chemotherapy regimens may also help to reduce the dose and therefore cumulative toxicities of cytotoxic chemotherapy.

### Clinical efficacy of targeted AA in combination with chemotherapy

#### Clinical studies of combination approaches in solid tumors

Outside the field of sarcoma, AA have been combined with chemotherapy with varying outcomes. A retrospective study of patients with advanced solid malignancies treated on phase 1 protocols between 2004 and 2013 showed that chemotherapy concomitant with VEGF(R) inhibitors was associated with significantly higher odds ratio for clinical benefit compared with chemotherapy without VEGF(R) inhibitors [32].

For example, in lung, breast, and colorectal carcinoma, AA have shown increased activity when combined with standard chemotherapy, as highlighted below. In advanced non-small cell lung cancer, a randomized phase 2 trial showed a trend towards increased response rate and time to progression when bevacizumab was combined with paclitaxel and carboplatin [33]. Several large randomized trials in patients with metastatic breast cancer showed significantly higher response rates and increased progression-free survival (PFS) when treated with bevacizumab combined with chemotherapy compared to those treated with chemotherapy alone [34–38].

Perhaps the disease in which bevacizumab has had the greatest impact in combination with chemotherapy is metastatic colorectal cancer. After a randomized phase 2 study showed encouraging results when bevacizumab was combined with fluorouracil and leucovorin [39], a randomized phase 3 trial of irinotecan, fluorouracil, and leucovorin with bevacizumab or placebo showed that bevacizumab increased response rate, time to progression, and overall survival [40]. Given these findings, bevacizumab is now included in the first-line management of patients with metastatic colorectal cancer. These clinical findings provided proof of principle of additive activity when AA are added to chemotherapy in patients with cancer and support clinical investigation in sarcoma.

#### Clinical studies of combination approaches in sarcoma

Targeted AA and chemotherapy have been combined in numerous early phase clinical trials in children and adults with advanced solid tumors. Phase 1 studies that included patients with sarcoma are summarized in Table 2. The backbone chemotherapy regimens used in these trials included taxane- and platinum-based therapies, camptothecins, and gemcitabine. Although not powered to evaluate the antitumor activity of AA combined with chemotherapy, the results of these phase 1 studies suggest that these regimens are generally well tolerated with promising clinical activity in sarcomas. In a phase 1b study of the combination of bevacizumab added to gemcitabine and docetaxel in patients with advanced STS, the overall response rate observed was 31 %, with 5 complete and 6 partial responses, and 18 patients with stable disease lasting for a median of 6 months [41]. Several pediatric phase 1 clinical trials have demonstrated the safety of combining AA, specifically bevacizumab, with cytotoxic chemotherapy in patients with advanced solid tumors, with tumor responses in patients with Ewing sarcoma [42]. In addition to those listed in Table 2, combination antiangiogenic approaches combining AA and conventional chemotherapy, such as ifosfamide and doxorubicin, studied in other malignancies, may warrant further study in sarcoma [43, 44].

**Table 2:** Completed phase 1 (or pilot) trials of combination approaches that enrolled patients with sarcoma

Drug combination	Sarcoma tumor type (number enrolled)	Responses^a^	Reference
*Trials with bevacizumab*			
Bevacizumab + pegylated SN-38 (EZN-2208)	STS (5)	SD (2)	[62]
Bevacizumab + bendamustine	Angiosarcoma (1)	None	[63]
Bevacizumab + irinotecan	RMS (1)	None	[64]
Bevacizumab + vincristine/irinotecan/temozolomide	STS (3); OS (2); ES (1)	SD (2)	[65]
Bevacizumab + vincristine/irinotecan/temozolomide	ES (2); RMS (1); Clear cell sarcoma (1)	CR (1); PR (1)	[42]
Bevacizumab + sorafenib + cyclophosphamide	OS (2); RMS (2); Other STS (4)	PR (1); SD (3)	[66]
Bevacizumab + gemcitabine/doxetaxel	STS (36)	CR (5); PR (6); SD (18)	[41]
Bevacizumab + ifosphamide/etoposide/carboplatin	STS (7); OS (3); Chondrosarcoma (2); Undifferentiated (1)	PR (4); SD (5)	[67]
*Trials with VEGFR and PDGFR inhibitors*			
Pazopanib + cisplatin	Sarcoma (5)	CR (1); SD (2)	[68]
Pazopanib + topotecan	STS (6); OS (2)	Unknown	[69]
Pazopanib + ifosfamide	Sarcoma (19)	PR (3)	[70]
Pazopanib + paclitaxel/carboplatin	OS (1); Giant cell tumor (1); Other sarcoma (1)	None	[71]
PDGFR inhibitor (CP-868,596) + docetaxel ± axitinib	ES (3); Other sarcoma (5)	SD (3)	[72]
Semaxanib + cisplatin/irinotecan	GIST (2); STS (1)	None	[73]
Sorafenib + irinotecan	OS (4); Synovial sarcoma (1); DSRCT (1); MPNST (1)	Unknown	[74]
Sunitinib + pemetrexed/carboplatin	Synovial sarcoma (1)	None	[75]
Sunitinib + gemcitabine	OS (1); STS (1)	SD (1)	[76]
Sunitinib + ifosfamide	ES (2); STS (6); Other sarcoma (7)	PR (2); SD (3)	[77]
Sunitinib + irinotecan	OS (1); STS (1)	None	[78]
Sunitinib + docetaxel	OS and STS (unknown)	None	[79]
*Trials with other antiangiogenic agents*			
Ombrabulin (AVE8062) + docetaxel	Muscle/bone tumors (5)	None	[80]
Thrombospondin-1 mimetic (ABT-510) + gemcitabine/cisplatin	Sarcoma (1)	None	[81]
Thrombospondin-1 mimetic (ABT-510) + 5-FU/leucovorin	Synovial sarcoma (1)	None	[82]
TNP-470 + paclitaxel/carboplatin	Sarcoma (2)	None	[83]

^a^Only includes SD, PR, and CR responses among patients with sarcoma. *CR* complete response; *DRSCT* desmoplastic small round cell tumor; *ES* Ewing sarcoma; *GIST* gastrointestinal stromal tumor; *MPNST* malignant peripheral nerve sheath tumor; *OS* osteosarcoma; *PDGFR* platelet-derived growth factor receptor; *PR* partial response; *RMS* rhabdomyosarcoma; *STS* soft tissue sarcoma; *SD* stable disease; *VEGF(R)* vascular endothelial growth factor (receptor)

There have been four reported phase 2 studies evaluating the combination of AA with chemotherapy in sarcoma. The combination of bevacizumab with doxorubicin was evaluated in 17 patients with metastatic STS [45]. While two partial responses (12 %) were observed, this response rate was not greater than that observed for single-agent doxorubicin [45]. However, 11 patients (65 %) had stable disease lasting four cycles or longer, suggesting that further consideration of this treatment regimen may be warranted in STS [45]. In general, the toxicity of bevacizumab and doxorubicin was similar to that reported for single-agent doxorubicin with one notable exception: the reported 35 % rate of grade 2 or higher cardiotoxicity with this combination regimen was greater than expected (compared to historical controls) [45]. Despite close monitoring and standard use of dexrazoxane, the observed cardiac toxicity warrants a change in the dose and/or schedule in future studies of this combination.

The Children’s Oncology Group (COG) evaluated bevacizumab or temsirolimus in combination with vinorelbine (V) and cyclophosphamide (C) in a randomized phase 2 study in patients with advanced rhabdomyosarcoma. Both treatment regimens were well tolerated and without unexpected toxicities. In a preliminary report, patients randomized to VC plus temsirolimus had a superior event-free survival compared to VC plus bevacizumab (65 vs. 50 %, respectively) [46]. As a VC alone arm was not included in the trial, it is not known if bevacizumab improved outcomes compared to the VC backbone.

Ray-Coquard and colleagues examined the addition of bevacizumab added to paclitaxel in a randomized phase 2 study of patients with angiosarcoma. While the combination antiangiogenic regimen was shown to be active in patients with angiosarcoma, the PFS and overall survival was similar in both arms [47]. Nevertheless, there was increased toxicity in the bevacizumab arm, which included one fatal drug-related toxicity (intestinal obstruction) [47]. The lack of benefit from bevacizumab may be due in part to key mutations in angiosarcoma that may activate the proangiogenic pathway independently of the classic ligand-receptor activation shown in recent studies. These findings suggest that the extracellular blockade of VEGF by a monoclonal antibody, such as bevacizumab, would not interfere with angiosarcoma proliferation [47]. Given these findings, the authors did not recommend the addition of bevacizumab to paclitaxel for the treatment of advanced angiosarcoma.

Recently, the Spanish Group for Research on Sarcomas presented their findings of a phase 2 study of sorafenib and ifosfamide in 35 patients with advanced STS [48]. This combination antiangiogenic regimen had acceptable toxicity in patients previously treated with anthracyclines. The study met its primary endpoint requiring at least 19/35 patients to be free of progression at 3 months. The combination was shown to be active in patients with advanced STS. Six (17 %) patients had partial responses to this regimen. The 3-month PFS was found to be 66 % (23/35) in patients treated with sorafenib plus ifosfamide, which may exceed the 3-month PFS in patients treated with ifosfamide alone, thus warranting further investigation [48].

Additional clinical trials evaluating combination therapy with targeted AA and cytotoxic chemotherapy in patients with sarcoma are ongoing (Table 3). With early promising results, the latest phase 2 trials have been largely directed towards pediatric sarcoma. These include bevacizumab, cyclophosphamide, and topotecan in patients with relapsed/refractory Ewing sarcoma (NCT01492673); and maintenance bevacizumab therapy in high-risk Ewing sarcoma and desmoplastic small round cell tumor (NCT01946529). Furthermore, the COG is actively enrolling patients on a randomized phase 2/3 trial of preoperative chemoradiation or preoperative radiation plus or minus pazopanib in STS histologies other than rhabdomyosarcoma (NCT02180867).

**Table 3:** Ongoing phase 1 (or pilot) clinical trials of combination approaches in sarcoma

Targeted antiangiogenic agent	Chemotherapy regimen	Tumor type	NCT
Bevacizumab	Doxorubicin/temsirolimus	Advanced solid tumors, including sarcoma	00761644
Bevacizumab	Doxorubicin	Advanced Kaposi sarcoma	00923936
Bevacizumab	Gemcitabine/docetaxel/valproic acid	Advanced sarcoma	01106872
Bevacizumab	Gemcitabine/paclitaxel	Advanced solid tumors, including sarcoma	01113476
Bevacizumab	Irinotecan/temozolomide + standard alkylator-based chemotherapy	Newly diagnosed DSRCT	01189643
Bevacizumab	Metronomic doxorubicin + radiation	Resectable STS	01746238
Bevacizumab	Metronomic cyclophosphamide/valproic acid/temsirolimus	Advanced solid tumors, including sarcoma	02446431
Pazopanib	Gemcitabine	Advanced leiomyosarcoma	01442662
Pazopanib	Docetaxel/gemcitabine	Operable STS	01719302
Pazopanib	Metronomic topotecan	Advanced solid tumors, including sarcoma	02303028

*DSRCT* desmoplastic small round cell tumor; *NCT* ClinicalTrials.gov Identifier/Number; *STS* soft tissue sarcoma

In adults, phase 2 studies are evaluating pazopanib and topotecan in patients with high-risk sarcomas (NCT02357810); pazopanib plus gemcitabine in advanced STS (NCT02203760, NCT01593748 and NCT01532687); pazopanib and paclitaxel in advanced angiosarcoma (NCT02212015); sorafenib, epirubicin, ifosfamide, and radiotherapy followed by surgery in high-risk STS (NCT02050919). Lastly, there is one open randomized phase 3 trial evaluating bevacizumab versus placebo combined with docetaxel and gemicitabine in the treatment of advanced uterine leiomyosarcoma (NCT01012297).

Outside of the context of formal clinical trials, several retrospective case studies/series have also highlighted the potential efficacy of these combination regimens. A child with transformed malignant angiosarcoma was successfully treated with bevacizumab, gemcitabine, and docetaxel, which resulted in temporary tumor regression with progression free survival of 12 months [49]. Dramatic improvement was also seen in another patient with inoperable face and neck angiosarcoma who was treated with bevacizumab and paclitaxel [50]. In three pediatric patients with Ewing sarcoma or undifferentiated sarcoma who were treated with bevacizumab, gemcitabine, and docetaxel, two patients had a partial response and the third patient had stable disease for >6 months [51]. Lastly, in a retrospective analysis of 14 patients with hemangiopericytomas and malignant solitary fibrous tumors who were treated with bevacizmuab and temozolomide, 11 patients (79 %) achieved a partial response, with a median time to response of 2.5 months [52].

Extensively reviewed elsewhere [53, 54], metronomic chemotherapy is an alternative antiangiogenic strategy, involving the application of daily, low-dose chemotherapy. With this low-dose approach, apoptosis is induced in the less frequently dividing endothelial cells rather than in the tumor cells [53]. This approach has been used in sarcoma with promising results [55–58]. In a feasibility study of metronomic cyclophosphamide plus prednisolone in 26 elderly patients with inoperable or metastatic STS, the response rate was 27 % and the disease control rate (responses and stable disease >12 weeks) was 69 % [56]. Currently, there are three open phase 1 studies examining the combination of bevacizumab or pazopanib added to metronomic chemotherapy that may include eligible sarcoma patients (Table 3).

## Conclusions

Advances in the biology of sarcomas have established the critical role of tumor angiogenesis and multiple signaling pathways involved in tumor development, growth, and therapy resistance. Numerous preclinical studies have demonstrated that targeting proangiogenic mechanisms in combination with cytotoxic chemotherapy may provide a valid approach to overcoming chemoresistance and inhibiting growth of these tumors. Early clinical data are still inconclusive, but some reports suggest that the use of these AA in combination with chemotherapy may be beneficial in the treatment of patients with advanced sarcoma.

Similar to various targeted therapeutic approaches that looked straightforward initially, antiangiogenesis has turned out to be more complex and nuanced than originally thought. Although VEGF seems to have a critical role in angiogenesis, our knowledge of the other molecular determinants of angiogenesis is still in its infancy. In fact, many of these pro- and antiangiogenic molecules are context- and dose-dependent. Additional studies are needed to understand these mechanisms and expand these findings to determine how to optimize these strategies for use in the management of patients with sarcoma. Ultimately, randomized studies are needed to demonstrate the benefit of angiogenesis inhibitors combined with chemotherapy.

## Abbreviations

AA: antiangiogenic agents
COG: Children’s Oncology Group
EGFR: epidermal growth factor receptor
ERK: extracellular signal-regulated kinase
IFP: interstitial fluid pressure
MAPK: mitogen-activated protein kinase
MVD: microvessel density
NSCLC: non-small cell lung cancer
PET: positron emission tomography
PDGFR: platelet-derived growth factor receptor
PFS: progression-free survival
STS: soft tissue sarcoma
VEGF(R): vascular endothelial growth factor (receptor)

## References

[1] Ganjoo K, Jacobs C (2010). Antiangiogenesis agents in the treatment of soft tissue sarcomas. Cancer.

[2] DuBois S, Demetri G (2007). Markers of angiogenesis and clinical features in patients with sarcoma. Cancer.

[3] DuBois SG, Marina N, Glade-Bender J (2010). Angiogenesis and vascular targeting in Ewing sarcoma: a review of preclinical and clinical data. Cancer.

[4] Kerbel RS (1991). Inhibition of tumor angiogenesis as a strategy to circumvent acquired resistance to anti-cancer therapeutic agents. BioEssays.

[5] Boehm T, Folkman J, Browder T, O’Reilly MS (1997). Antiangiogenic therapy of experimental cancer does not induce acquired drug resistance. Nature.

[6] Jain RK (2014). Antiangiogenesis strategies revisited: from starving tumors to alleviating hypoxia. Cancer Cell.

[7] Sleijfer S, Ray-Coquard I, Papai Z, Le Cesne A, Scurr M, Schoffski P, Collin F, Pandite L, Marreaud S, De Brauwer A (2009). Pazopanib, a multikinase angiogenesis inhibitor, in patients with relapsed or refractory advanced soft tissue sarcoma: a phase II study from the European organisation for research and treatment of cancer-soft tissue and bone sarcoma group (EORTC study 62043). J Clin Oncol.

[8] Carmeliet P, Jain RK (2000). Angiogenesis in cancer and other diseases. Nature.

[9] Liekens S, De Clercq E, Neyts J (2001). Angiogenesis: regulators and clinical applications. Biochem Pharmacol.

[10] Goel S, Duda DG, Xu L, Munn LL, Boucher Y, Fukumura D, Jain RK (2011). Normalization of the vasculature for treatment of cancer and other diseases. Physiol Rev.

[11] Tong RT, Boucher Y, Kozin SV, Winkler F, Hicklin DJ, Jain RK (2004). Vascular normalization by vascular endothelial growth factor receptor 2 blockade induces a pressure gradient across the vasculature and improves drug penetration in tumors. Cancer Res.

[12] Winkler F, Kozin SV, Tong RT, Chae SS, Booth MF, Garkavtsev I, Xu L, Hicklin DJ, Fukumura D, di Tomaso E (2004). Kinetics of vascular normalization by VEGFR2 blockade governs brain tumor response to radiation: role of oxygenation, angiopoietin-1, and matrix metalloproteinases. Cancer Cell.

[13] Yuan F, Chen Y, Dellian M, Safabakhsh N, Ferrara N, Jain RK (1996). Time-dependent vascular regression and permeability changes in established human tumor xenografts induced by an anti-vascular endothelial growth factor/vascular permeability factor antibody. Proc Natl Acad Sci USA.

[14] Liu Y, Xia X, Zhou M, Liu X (2015). Avastin(R) in combination with gemcitabine and cisplatin significantly inhibits tumor angiogenesis and increases the survival rate of human A549 tumor-bearing mice. Exp Ther Med.

[15] Dickson PV, Hamner JB, Sims TL, Fraga CH, Ng CY, Rajasekeran S, Hagedorn NL, McCarville MB, Stewart CF, Davidoff AM (2007). Bevacizumab-induced transient remodeling of the vasculature in neuroblastoma xenografts results in improved delivery and efficacy of systemically administered chemotherapy. Clin Cancer Res.

[16] Vangestel C, Van de Wiele C, Van Damme N, Staelens S, Pauwels P, Reutelingsperger CP, Peeters M (2011). (99)mTc-(CO)(3) His-annexin A5 micro-SPECT demonstrates increased cell death by irinotecan during the vascular normalization window caused by bevacizumab. J Nucl Med.

[17] Turley RS, Fontanella AN, Padussis JC, Toshimitsu H, Tokuhisa Y, Cho EH, Hanna G, Beasley GM, Augustine CK, Dewhirst MW (2012). Bevacizumab-induced alterations in vascular permeability and drug delivery: a novel approach to augment regional chemotherapy for in-transit melanoma. Clin Cancer Res.

[18] Chatterjee S, Wieczorek C, Schottle J, Siobal M, Hinze Y, Franz T, Florin A, Adamczak J, Heukamp LC, Neumaier B (2014). Transient antiangiogenic treatment improves delivery of cytotoxic compounds and therapeutic outcome in lung cancer. Cancer Res.

[19] Van der Veldt AA, Lubberink M, Bahce I, Walraven M, de Boer MP, Greuter HN, Hendrikse NH, Eriksson J, Windhorst AD, Postmus PE (2012). Rapid decrease in delivery of chemotherapy to tumors after anti-VEGF therapy: implications for scheduling of anti-angiogenic drugs. Cancer Cell.

[20] Zhou Q, Gallo JM (2009). Differential effect of sunitinib on the distribution of temozolomide in an orthotopic glioma model. Neuro Oncol.

[21] Zhou Q, Guo P, Gallo JM (2008). Impact of angiogenesis inhibition by sunitinib on tumor distribution of temozolomide. Clin Cancer Res.

[22] Paez-Ribes M, Man S, Xu P, Kerbel RS (2015). Potential proinvasive or metastatic effects of preclinical antiangiogenic therapy are prevented by concurrent chemotherapy. Clin Cancer Res.

[23] Li Y, Yang X, Su LJ, Flaig TW (2011). Pazopanib synergizes with docetaxel in the treatment of bladder cancer cells. Urology..

[24] Augustine CK, Toshimitsu H, Jung SH, Zipfel PA, Yoo JS, Yoshimoto Y, Selim MA, Burchette J, Beasley GM, McMahon N (2010). Sorafenib, a multikinase inhibitor, enhances the response of melanoma to regional chemotherapy. Mol Cancer Ther.

[25] Troiani T, Lockerbie O, Morrow M, Ciardiello F, Eckhardt SG (2006). Sequence-dependent inhibition of human colon cancer cell growth and of prosurvival pathways by oxaliplatin in combination with ZD6474 (Zactima), an inhibitor of VEGFR and EGFR tyrosine kinases. Mol Cancer Ther.

[26] Naumova E, Ubezio P, Garofalo A, Borsotti P, Cassis L, Riccardi E, Scanziani E, Eccles SA, Bani MR, Giavazzi R (2006). The vascular targeting property of paclitaxel is enhanced by SU6668, a receptor tyrosine kinase inhibitor, causing apoptosis of endothelial cells and inhibition of angiogenesis. Clin Cancer Res.

[27] Humbert M, Casteran N, Letard S, Hanssens K, Iovanna J, Finetti P, Bertucci F, Bader T, Mansfield CD, Moussy A (2010). Masitinib combined with standard gemcitabine chemotherapy: in vitro and in vivo studies in human pancreatic tumour cell lines and ectopic mouse model. PLoS One.

[28] Zhang L, Yu D, Hicklin DJ, Hannay JA, Ellis LM, Pollock RE (2002). Combined anti-fetal liver kinase 1 monoclonal antibody and continuous low-dose doxorubicin inhibits angiogenesis and growth of human soft tissue sarcoma xenografts by induction of endothelial cell apoptosis. Cancer Res.

[29] Zhang L, Hannay JA, Liu J, Das P, Zhan M, Nguyen T, Hicklin DJ, Yu D, Pollock RE, Lev D (2006). Vascular endothelial growth factor overexpression by soft tissue sarcoma cells: implications for tumor growth, metastasis, and chemoresistance. Cancer Res.

[30] Kumar S, Mokhtari RB, Sheikh R, Wu B, Zhang L, Xu P, Man S, Oliveira ID, Yeger H, Kerbel RS (2011). Metronomic oral topotecan with pazopanib is an active antiangiogenic regimen in mouse models of aggressive pediatric solid tumor. Clin Cancer Res.

[31] Ren W, Korchin B, Lahat G, Wei C, Bolshakov S, Nguyen T, Merritt W, Dicker A, Lazar A, Sood A (2008). Combined vascular endothelial growth factor receptor/epidermal growth factor receptor blockade with chemotherapy for treatment of local, uterine, and metastatic soft tissue sarcoma. Clin Cancer Res.

[32] Tang C, Hess K, Jardim DL, Gagliato Dde M, Tsimberidou AM, Falchook G, Fu S, Janku F, Naing A, Piha-Paul S (2014). Synergy between VEGF/VEGFR inhibitors and chemotherapy agents in the phase I clinic. Cancer Res.

[33] Johnson DH, Fehrenbacher L, Novotny WF, Herbst RS, Nemunaitis JJ, Jablons DM, Langer CJ, DeVore RF, Gaudreault J, Damico LA (2004). Randomized phase II trial comparing bevacizumab plus carboplatin and paclitaxel with carboplatin and paclitaxel alone in previously untreated locally advanced or metastatic non-small-cell lung cancer. J Clin Oncol.

[34] Miller KD, Chap LI, Holmes FA, Cobleigh MA, Marcom PK, Fehrenbacher L, Dickler M, Overmoyer BA, Reimann JD, Sing AP (2005). Randomized phase III trial of capecitabine compared with bevacizumab plus capecitabine in patients with previously treated metastatic breast cancer. J Clin Oncol.

[35] Miller K, Wang M, Gralow J, Dickler M, Cobleigh M, Perez EA, Shenkier T, Cella D, Davidson NE (2007). Paclitaxel plus bevacizumab versus paclitaxel alone for metastatic breast cancer. N Engl J Med.

[36] Pivot X, Schneeweiss A, Verma S, Thomssen C, Passos-Coelho JL, Benedetti G, Ciruelos E, von Moos R, Chang HT, Duenne AA (2011). Efficacy and safety of bevacizumab in combination with docetaxel for the first-line treatment of elderly patients with locally recurrent or metastatic breast cancer: results from AVADO. Eur J Cancer.

[37] Robert NJ, Dieras V, Glaspy J, Brufsky AM, Bondarenko I, Lipatov ON, Perez EA, Yardley DA, Chan SY, Zhou X (2011). RIBBON-1: randomized, double-blind, placebo-controlled, phase III trial of chemotherapy with or without bevacizumab for first-line treatment of human epidermal growth factor receptor 2-negative, locally recurrent or metastatic breast cancer. J Clin Oncol.

[38] Pignata S, Lorusso D, Scambia G, Sambataro D, Tamberi S, Cinieri S, Mosconi AM, Orditura M, Brandes AA, Arcangeli V (2015). Pazopanib plus weekly paclitaxel versus weekly paclitaxel alone for platinum-resistant or platinum-refractory advanced ovarian cancer (MITO 11): a randomised, open-label, phase 2 trial. Lancet Oncol.

[39] Kabbinavar F, Hurwitz HI, Fehrenbacher L, Meropol NJ, Novotny WF, Lieberman G, Griffing S, Bergsland E (2003). Phase II, randomized trial comparing bevacizumab plus fluorouracil (FU)/leucovorin (LV) with FU/LV alone in patients with metastatic colorectal cancer. J Clin Oncol.

[40] Hurwitz H, Fehrenbacher L, Novotny W, Cartwright T, Hainsworth J, Heim W, Berlin J, Baron A, Griffing S, Holmgren E (2004). Bevacizumab plus irinotecan, fluorouracil, and leucovorin for metastatic colorectal cancer. N Engl J Med.

[41] Verschraegen CF, Arias-Pulido H, Lee SJ, Movva S, Cerilli LA, Eberhardt S, Schmit B, Quinn R, Muller CY, Rabinowitz I (2012). Phase IB study of the combination of docetaxel, gemcitabine, and bevacizumab in patients with advanced or recurrent soft tissue sarcoma: the Axtell regimen. Ann Oncol.

[42] Wagner L, Turpin B, Nagarajan R, Weiss B, Cripe T, Geller J (2013). Pilot study of vincristine, oral irinotecan, and temozolomide (VOIT regimen) combined with bevacizumab in pediatric patients with recurrent solid tumors or brain tumors. Pediatr Blood Cancer.

[43] Hainsworth JD, Firdaus ID, Earwood CB, Chua CC (2015). Pazopanib and liposomal doxorubicin in the treatment of patients with relapsed/refractory epithelial ovarian cancer: a phase Ib study of the Sarah Cannon Research Institute. Cancer Invest.

[44] Vergote I, Schilder RJ, Pippitt CH, Wong S, Gordon AN, Scudder S, Kridelka F, Dirix L, Leach JW, Ananda S (2014). A phase 1b study of trebananib in combination with pegylated liposomal doxorubicin or topotecan in women with recurrent platinum-resistant or partially platinum-sensitive ovarian cancer. Gynecol Oncol.

[45] D’Adamo DR, Anderson SE, Albritton K, Yamada J, Riedel E, Scheu K, Schwartz GK, Chen H, Maki RG (2005). Phase II study of doxorubicin and bevacizumab for patients with metastatic soft-tissue sarcomas. J Clin Oncol.

[46] Mascarenhas L, Meyers WH, Lyden E, Rodeberg DA (2014). Randomized phase II trial of bevacizumab and temsirolimus in combination with vinorelbine (V) and cyclophosphamide (C) for first relapse/disease progression of rhabdomyosarcoma (RMS): a report from the Children’s Oncology Group (COG). J Clin Oncol.

[47] Ray-Coquard IL, Domont J, Tresch-Bruneel E, Bompas E, Cassier PA, Mir O, Piperno-Neumann S, Italiano A, Chevreau C, Cupissol D (2015). Paclitaxel given once per week with or without bevacizumab in patients with advanced angiosarcoma: a randomized phase ii trial. J Clin Oncol.

[48] Del Muro XG, Maurel J, Trufero JM, Lavernia J, Lopez-Pousa A, De Las Penas R, Cubedo R, Fra J, Casado A, De Juan A et al. Phase II trial of ifosfamide in combination with sorafenib in patients with advanced soft tissue sarcoma: A Spanish Group for Research on Sarcomas (GEIS) study. J Clin Oncol. 2013; 31. **(suppl; abstr 10523)**.

[49] Jeng MR, Fuh B, Blatt J, Gupta A, Merrow AC, Hammill A, Adams D (2014). Malignant transformation of infantile hemangioma to angiosarcoma: response to chemotherapy with bevacizumab. Pediatr Blood Cancer.

[50] Fuller CK, Charlson JA, Dankle SK, Russell TJ (2010). Dramatic improvement of inoperable angiosarcoma with combination paclitaxel and bevacizumab chemotherapy. J Am Acad Dermatol.

[51] Hingorani P, Eshun F, White-Collins A, Watanabe M (2012). Gemcitabine, docetaxel, and bevacizumab in relapsed and refractory pediatric sarcomas. J Pediatr Hematol Oncol.

[52] Park MS, Patel SR, Ludwig JA, Trent JC, Conrad CA, Lazar AJ, Wang WL, Boonsirikamchai P, Choi H, Wang X (2011). Activity of temozolomide and bevacizumab in the treatment of locally advanced, recurrent, and metastatic hemangiopericytoma and malignant solitary fibrous tumor. Cancer.

[53] Kerbel RS, Kamen BA (2004). The anti-angiogenic basis of metronomic chemotherapy. Nat Rev Cancer.

[54] Penel N, Adenis A, Bocci G (2012). Cyclophosphamide-based metronomic chemotherapy: after 10 years of experience, where do we stand and where are we going?. Crit Rev Oncol Hematol.

[55] Felgenhauer JL, Nieder ML, Krailo MD, Bernstein ML, Henry DW, Malkin D, Baruchel S, Chuba PJ, Sailer SL, Brown K (2013). A pilot study of low-dose anti-angiogenic chemotherapy in combination with standard multiagent chemotherapy for patients with newly diagnosed metastatic Ewing sarcoma family of tumors: a Children’s Oncology Group (COG) Phase II study NCT00061893. Pediatr Blood Cancer.

[56] Mir O, Domont J, Cioffi A, Bonvalot S, Boulet B, Le Pechoux C, Terrier P, Spielmann M, Le Cesne A (2011). Feasibility of metronomic oral cyclophosphamide plus prednisolone in elderly patients with inoperable or metastatic soft tissue sarcoma. Eur J Cancer.

[57] Italiano A, Toulmonde M, Lortal B, Stoeckle E, Garbay D, Kantor G, Kind M, Coindre JM, Bui B (2010). “Metronomic” chemotherapy in advanced soft tissue sarcomas. Cancer Chemother Pharmacol.

[58] Briasoulis E, Pappas P, Puozzo C, Tolis C, Fountzilas G, Dafni U, Marselos M, Pavlidis N (2009). Dose-ranging study of metronomic oral vinorelbine in patients with advanced refractory cancer. Clin Cancer Res.

[59] Dalal S, Burchill SA (2009). Preclinical evaluation of vascular-disrupting agents in Ewing’s sarcoma family of tumours. Eur J Cancer.

[60] Vistica DT, Hollingshead M, Borgel SD, Kenney S, Stockwin LH, Raffeld M, Schrump DS, Burkett S, Stone G, Butcher DO (2009). Therapeutic vulnerability of an in vivo model of alveolar soft part sarcoma (ASPS) to antiangiogenic therapy. J Pediatr Hematol Oncol.

[61] Ma G, Masuzawa M, Hamada Y, Haraguchi F, Tamauchi H, Sakurai Y, Fujimura T, Katsuoka K (2000). Treatment of murine angiosarcoma with etoposide, TNP-470 and prednisolone. J Dermatol Sci.

[62] Jeong W, Park SR, Rapisarda A, Fer N, Kinders RJ, Chen A, Melillo G, Turkbey B, Steinberg SM, Choyke P (2014). Weekly EZN-2208 (PEGylated SN-38) in combination with bevacizumab in patients with refractory solid tumors. Invest New Drugs.

[63] Tsimberidou AM, Adamopoulos AM, Ye Y, Piha-Paul S, Janku F, Fu S, Hong D, Falchook GS, Naing A, Wheler J (2014). Phase I clinical trial of bendamustine and bevacizumab for patients with advanced cancer. J Natl Compr Canc Netw.

[64] Okada K, Yamasaki K, Tanaka C, Fujisaki H, Osugi Y, Hara J (2013). Phase I study of bevacizumab plus irinotecan in pediatric patients with recurrent/refractory solid tumors. Jpn J Clin Oncol.

[65] Venkatramani R, Malogolowkin M, Davidson TB, May W, Sposto R, Mascarenhas L (2013). A phase I study of vincristine, irinotecan, temozolomide and bevacizumab (vitb) in pediatric patients with relapsed solid tumors. PLoS One.

[66] Navid F, Baker SD, McCarville MB, Stewart CF, Billups CA, Wu J, Davidoff AM, Spunt SL, Furman WL, McGregor LM (2013). Phase I and clinical pharmacology study of bevacizumab, sorafenib, and low-dose cyclophosphamide in children and young adults with refractory/recurrent solid tumors. Clin Cancer Res.

[67] Jordan K, Wolf HH, Voigt W, Kegel T, Mueller LP, Behlendorf T, Sippel C, Arnold D, Schmoll HJ (2010). Bevacizumab in combination with sequential high-dose chemotherapy in solid cancer, a feasibility study. Bone Marrow Transpl.

[68] Dieras V, Bachelot T, Campone M, Isambert N, Joly F, LeTourneau C, Cassier PA, Bompas E, Fumoleau P, Noal S (2014). Pazopanib (P) and cisplatin (CDDP) in patients with advanced solid tumors: a UNICANCER phase I study. J Clin Oncol.

[69] Kerklaan BM, Lolkema MP, Devriese LA, Voest EE, Nol-Boekel A, Mergui-Roelvink M, Mykulowycz K, Stoebenau JE, Fang L, Legenne P (2013). Phase I study of safety, tolerability, and pharmacokinetics of pazopanib in combination with oral topotecan in patients with advanced solid tumors. J Clin Oncol.

[70] Hamberg P, Boers-Sonderen MJ, van der Graaf WT, de Bruijn P, Suttle AB, Eskens FA, Verweij J, van Herpen CM, Sleijfer S (2014). Pazopanib exposure decreases as a result of an ifosfamide-dependent drug-drug interaction: results of a phase I study. Br J Cancer.

[71] Burris HA, Dowlati A, Moss RA, Infante JR, Jones SF, Spigel DR, Levinson KT, Lindquist D, Gainer SD, Dar MM (2012). Phase I study of pazopanib in combination with paclitaxel and carboplatin given every 21 days in patients with advanced solid tumors. Mol Cancer Ther.

[72] Michael M, Vlahovic G, Khamly K, Pierce KJ, Guo F, Olszanski AJ (2010). Phase Ib study of CP-868,596, a PDGFR inhibitor, combined with docetaxel with or without axitinib, a VEGFR inhibitor. Br J Cancer.

[73] Martin LK, Bekaii-Saab T, Serna D, Monk P, Clinton SK, Grever MR, Kraut EH (2013). A phase I dose escalation and pharmacodynamic study of SU5416 (semaxanib) combined with weekly cisplatin and irinotecan in patients with advanced solid tumors. Onkologie.

[74] Meany HJ, Dome J, Hinds PS, Bagatell R, Shusterman S, Widemann BC, Stern E, London WB, Kim A, Fox E (2014). Phase 1 study of sorafenib and irinotecan in pediatric patients with relapsed or refractory solid tumors. J Clin Oncol.

[75] Blais N, Camidge DR, Jonker DJ, Soulieres D, Laurie SA, Diab SG, Ruiz-Garcia A, Thall A, Zhang K, Chao RC (2013). Sunitinib combined with pemetrexed and carboplatin in patients with advanced solid malignancies–results of a phase I dose-escalation study. Invest New Drugs.

[76] Brell JM, Krishnamurthi SS, Rath L, Bokar JA, Savvides P, Gibbons J, Cooney MM, Meropol NJ, Ivy P, Dowlati A (2012). Phase I trial of sunitinib and gemcitabine in patients with advanced solid tumors. Cancer Chemother Pharmacol.

[77] Hamberg P, Steeghs N, Loos WJ, van de Biessen D, den Hollander M, Tascilar M, Verweij J, Gelderblom H, Sleijfer S (2010). Decreased exposure to sunitinib due to concomitant administration of ifosfamide: results of a phase I and pharmacokinetic study on the combination of sunitinib and ifosfamide in patients with advanced solid malignancies. Br J Cancer.

[78] Boven E, Massard C, Armand JP, Tillier C, Hartog V, Brega NM, Countouriotis AM, Ruiz-Garcia A, Soria JC (2010). A phase I, dose-finding study of sunitinib in combination with irinotecan in patients with advanced solid tumours. Br J Cancer.

[79] Robert F, Sandler A, Schiller JH, Liu G, Harper K, Verkh L, Huang X, Ilagan J, Tye L, Chao R (2010). Sunitinib in combination with docetaxel in patients with advanced solid tumors: a phase I dose-escalation study. Cancer Chemother Pharmacol.

[80] Eskens FA, Tresca P, Tosi D, Van Doorn L, Fontaine H, Van der Gaast A, Veyrat-Follet C, Oprea C, Hospitel M, Dieras V (2014). A phase I pharmacokinetic study of the vascular disrupting agent ombrabulin (AVE8062) and docetaxel in advanced solid tumours. Br J Cancer.

[81] Gietema JA, Hoekstra R, de Vos FY, Uges DR, van der Gaast A, Groen HJ, Loos WJ, Knight RA, Carr RA, Humerickhouse RA (2006). A phase I study assessing the safety and pharmacokinetics of the thrombospondin-1-mimetic angiogenesis inhibitor ABT-510 with gemcitabine and cisplatin in patients with solid tumors. Ann Oncol.

[82] Hoekstra R, de Vos FY, Eskens FA, de Vries EG, Uges DR, Knight R, Carr RA, Humerickhouse R, Verweij J, Gietema JA (2006). Phase I study of the thrombospondin-1-mimetic angiogenesis inhibitor ABT-510 with 5-fluorouracil and leucovorin: a safe combination. Eur J Cancer.

[83] Tran HT, Blumenschein GR, Lu C, Meyers CA, Papadimitrakopoulou V, Fossella FV, Zinner R, Madden T, Smythe LG, Puduvalli VK (2004). Clinical and pharmacokinetic study of TNP-470, an angiogenesis inhibitor, in combination with paclitaxel and carboplatin in patients with solid tumors. Cancer Chemother Pharmacol.

